# Follow‐Up of Left‐Ventricular Assist Device Patients With Telemonitoring: A National Retrospective Multicentric Study on the Satelia LVAD Web Application

**DOI:** 10.1111/aor.70038

**Published:** 2025-11-08

**Authors:** Clément Delmas, Anne‐Sophie Simoni, Céline Goeminne, Aude Boignard, Anne‐Céline Martin, Fabrice Vanhuyse, Erwan Flecher, Catherine Nafeh‐Bizet, Romain Itier, Nicolas Pages, Sophie Nisse‐Durgeat, Pascal Battistella, Karine Nubret‐le‐Coniat

**Affiliations:** ^1^ Cardiology Department Centre Hospitalier Universitaire de Toulouse Cardiologie Toulouse France; ^2^ Recherche et Enseignement en Insuffisance Cardiaque Avancée, Transplantation et Assistance (REICATRA) Institut Saint Jacques, Centre Hospitalier Universitaire de Toulouse Cardiologie Toulouse France; ^3^ Assistance Publique ‐ Hopitaux de Marseille Provence‐Alpes‐Côte D'azur Marseille France; ^4^ Centre Hospitalier Universitaire de Lille, Lille France; ^5^ Centre Hospitalier Universitaire Grenoble Alpes Grenoble, Auvergne‐Rhône‐Alpes Grenoble France; ^6^ Hopital Europeen Georges‐Pompidou Service de Cardiologie Paris France; ^7^ Centre Hospitalier Regional Universitaire de Nancy Nancy Grand Est France; ^8^ Division of Thoracic and Cardiovascular Surgery Univ Rennes, Centre Hospitalier Universitaire de Rennes Rennes Brittany France; ^9^ Thoracic and Cardiovascular Surgery Department Centre Hospitalier Universitaire de Rouen Rouen Normandy France; ^10^ NP Medical, Bordeaux Nouvelle‐ Aquitaine France; ^11^ Centre Hospitalier Universitaire Montpellier Montpellier Occitanie France; ^12^ Centre Hospitalier Universitaire de Bordeaux Centre Medico‐Chirurgical Magellan Pessac Nouvelle‐Aquitaine France

**Keywords:** advanced heart failure, follow‐ups, heart disease, LVADS, telemonitoring, ventricular assist devices

## Abstract

**Background:**

Left‐ventricular assist devices (LVADs) are a major therapeutic option in advanced heart failure (adHF), improving survival rates and quality of life (QoL). Complications, however, can alter their prognosis. Specialized telemonitoring could facilitate LVAD follow‐ups and improve outcomes. This study aimed to evaluate the usefulness of telemonitoring for LVAD patients.

**Methods:**

Patients, followed up with a web application (SateliaLVAD), were included in a national retrospective study at nine tertiary hospitals in France. Characteristics and detected hospitalization risk alerts data were collected. The risk of hospitalization was categorized based on a clinical algorithm (green: no risk, orange: heightened risk, and red: emergent contact with patient and possible hospitalization).

**Results:**

In total, 161 patients were included (male: 82.0%, mean age: 62.2 years). Indications for LVAD were mainly ischemic cardiomyopathy (82.0%) and bridge to transplant (50.3%). The mean follow‐up duration lasted 19.9 [1–45] months with 76 (47.2%) patients continuing telemonitoring. Compliance was high (79.0%). The main reason for cessation was death (30.6%). Total hospitalization risk alerts detected by telemonitoring were: orange alerts (*n* = 8265, 72.3%) and red alerts (*n* = 1613, 14.1%) with 48.5% of cases resolved (orange: 50.8% vs. red: 54.8%). The most frequent type of resolved alert was for a measured risk of cardiac decompensation (orange: 2227 vs. red: 382).

**Conclusion:**

To our knowledge, this is the first extensive study to describe the follow‐up of LVAD patients by a dedicated telemonitoring application. Telemonitoring as a specific follow‐up tool may be feasible for this subpopulation. Future randomized studies on specific prospective evaluations such as survival and QoL are needed.

## Graphical abstract

We evaluated the usefulness of remote monitoring for advanced heart failure patients using left‐ventricular assist devices (LVADs) in France. A national, retrospective study on a web application (Satelia) at nine locations was conducted. Specialized remote monitoring could facilitate LVAD follow‐ups and improve outcomes.

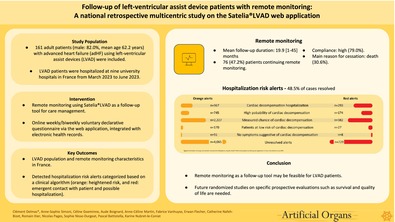

**Abbreviations**
adHF
: Advanced heart failure
BTT
: Bridge to transplant therapy
DT
: Destination therapy
HCP
: Healthcare professional
ICU
: Intensive Care Unit
LVAD
: Left‐ventricular assist device
LVEF
: Left Ventricular Ejection Fraction
NHYA
: New York Heart Association
NPV
: Negative Predictive Value
QoL
: Quality of Life

## 1 Background

Advanced heart failure (adHF) is an end‐stage heart failure disease with associated organ dysfunction and poor prognosis with a reported 1 year mortality of 30 to 48% [1, 2]. Patients with adHF account for an estimated 1 to 10% of the global heart failure population [3, 4]. Currently, adHF guideline‐directed medical therapies are less effective, with advanced therapies or palliative care being required [5, 6]. Moreover, therapeutic opportunities for adHF patients have increasingly focused on left‐ventricular assist devices (LVAD) regardless of being a destination therapy (DT) or bridge to transplant therapy (BTT) given the shortage of organs, longer waiting list times and risks of patients' worsening conditions [7, 8].

LVAD is considered on par with heart transplantation at two years with a reported 79% 2 year survival rate on device support [9]. Around 200 patients are treated with LVAD annually in France; however, literature on this population is limited [10]. To date, studies have shown that LVAD is particularly favorable as a BTT and improves the quality of life (QoL) of patients, even though survival rates are lower when compared to the US [11].

Monitoring of LVAD patients is necessary as a therapeutic intervention due to the specific complications and management of the devices themselves. Moreover, monitoring is considered part of the treatment since complications can be preventable or curable if applied on time. For ambulatory patients with adHF, one out of two patients survive either from an LVAD or a heart transplant within 2 years of a monitoring intervention [6]. For patients who are followed‐up by specialized tertiary centers, monitoring may serve both as a challenge and an opportunity to improve quality of care [12].

Telehealth tools such as telemonitoring systems can improve follow‐up outcomes, given the importance of self‐management and patient centricity in heart failure [13, 14]. The use of smart devices and applications has empowered patients with chronic diseases and has been reported to be an effective tool for healthcare professionals (HCPs) [12]. In France, telemonitoring systems are prescription based and are reimbursed by the national health insurance. Satelia Cardio is one such system based on a web application that is used for therapeutic education and monitoring of heart failure [15]. Even though it is a telehealth solution, its publicly funded program applies both human and digital interactions, and has provided care to over 8500 patients specifically for heart failure management since 2018. For LVAD patients, the web application has a specific algorithm to estimate the risk of hospitalizations and has an integrated alarms system to send messages and communicate with both patients and HCPs. Compared to the total population of patients receiving LVAD in France, approximately 20% of patients have been reported to use the telemonitoring system. The objective of this study was to identify and describe the LVAD population in France using Satelia LVAD (Satelia Cardio specifically personalized to LVAD patients) and evaluate the use of telemonitoring as a follow‐up tool for this specific subgroup of adHF patients.

## 2 Methods

### 2.1 Study Design

A national, retrospective, multicentric study was conducted at nine university hospitals from March 2023 to June 2023. An online weekly or biweekly voluntary declarative questionnaire was sent to patients to complete via the Satelia LVAD web application which was integrated with their electronic health records. Patients were initially registered for telemonitoring with their providing cardiologist and received a call for onboarding by a specialized nurse. Every week, patients were required to fill out the provided digital questionnaire and assess and record their weight. The web application used a score‐based algorithm to analyze the responses of the questionnaires which triggered alerts to, first, automatically adjust the follow‐up frequency toward the risk profile of a patient and, second, notify the cardiologist or nurse when there was a risk of cardiac decompensation. Interventions taken as a result of the alerts included having the cardiologist or nurse contact the patient regarding the symptoms declared online and determining whether the patients should have a consultation physical or online, or be hospitalized depending on the clinical degradation of their condition.

The web application was sectioned into four parts: (i) administrative file, (ii) patient file, (iii) patient monitoring, and (iv) key events (Table 1). The administrative file included medical, pharmacology, paramedical and patient contact information. Patient files comprised information including current treatments, comorbidities, LVEF, LVAD type, and heart disease status.

**TABLE 1:** Descriptions of the sections from the web application (Satelia LVAD).

Section	Description
Administrative file	Medical, pharmacology, paramedical and patient contact information
Patient file	Current treatments, comorbidities, LVEF, and heart disease status as well as transfer pictures of the transcutaneous line and prescriptions for medication and/or biological samples
Patient monitoring	Functional and specific questionnaires to LVAD patients sent 2 times a week Algorithm generating a score and alarms via SMS and/or email
Important events	Complications and hospitalizations detected by the clinical algorithm

The questionnaires used for monitoring patients were both functional and specific to LVADs. The questionnaire items were related to weight, heart failure symptoms (such as fatigue, edema, shortness of breath and coughing) as well as specific signs of LVAD‐related complications' signs such as vision disorders, hematuria, shortness of breath at rest, dizziness and headaches as well as a description of driveline orifice. Patients were able to send transfer pictures of the transcutaneous line, as well as prescriptions for medication and/or biological samples.

Monitoring of patients was standardized throughout the location sites and a universal script of the questionnaire was provided to all patients. Data from the questionnaires were directly entered by the patients or by their caregivers on a voluntary basis. Patients who could not use the application or who had poor digital literacy were called by a specialized nurse via phone to collect and fill out the questionnaire answers and provide the given weight of the patients. In addition to telemonitoring, patients received standard follow‐up care in accordance with the local practices and protocols of each participating center. This follow‐up involved physical consultations or day hospitalizations at least once every 3 months, during which clinical, laboratory, and echocardiographic assessments were performed. Depending on the center and individual patient needs, more frequent visitations and additional examinations could be arranged at the discretion of the medical team in charge. Further details on how the web application works were previously described in literature from its original form (Satelia Cardio) relating to heart failure, in which the questionnaires were validated [16, 17]. The Satelia LVAD questionnaire is provided in Table S1.

### 2.2 Inclusion/Exclusion Criteria

**FIGURE 1:**
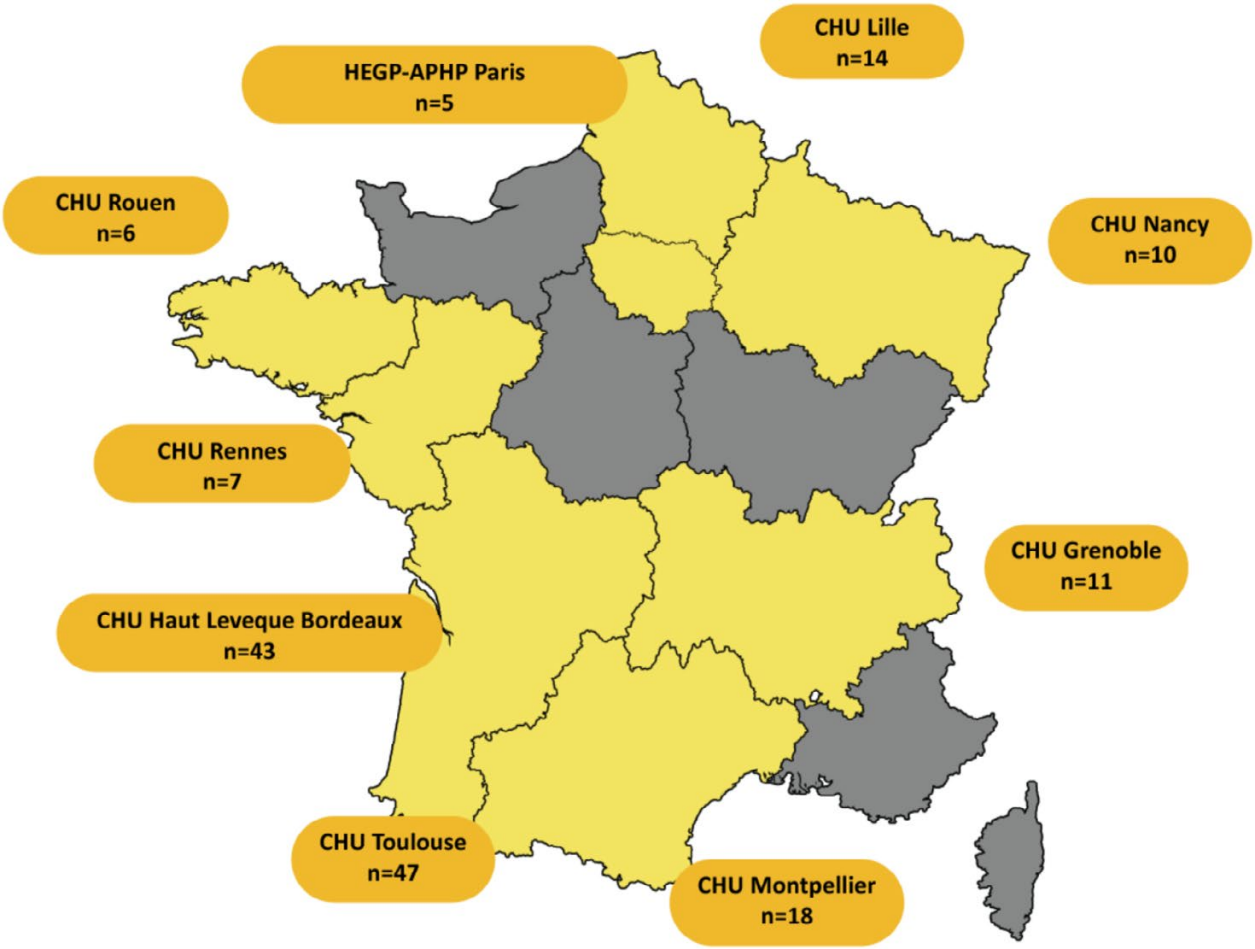
The location and number of participating LVAD patients using telemonitoring in France. *Orange alert: Raising awareness to the healthcare professionals and team for the patient to have a consultation (phone call or in‐person). **Red alert: Raising awareness for the team for emergent contact with patients and possible hospitalization. †Alerts were unresolved if the healthcare professional (primarily cardiologists) provided care to the patient, however, did not click resolved in the application or was not concerned by the detected alert because it was not relevant for the care of the patient. [Color figure can be viewed at wileyonlinelibrary.com]

Included participants were adHF patients with LVADs who: (i) resided in metropolitan France, (ii) had a prior hospitalization for heart failure and (iii) were followed up with the web application located in one of the 14 LVAD reference centers (Figure 1). There were no specific exclusion criteria; however, those that were not physically or mentally able to use telemonitoring and those that refused to participate in the program were excluded from the study.

### 2.3 Variables and Data Collection

Data collected included patient characteristics (age, sex and location of care) and medical characteristics such as weight, left ventricular ejection fraction (LVEF), NYHA classification, hypertension, heart failure etiology, cardiovascular risk factors, comorbidities, preimplantation and at inclusion parameters, BNP and NT‐proBNP, bilirubin, creatinine and eGFR levels as well as LVAD‐related items including the Tricuspid Annular Plane Systolic Excursion (TAPSE), reference power and flow, pulsatility index and speed (revolutions per minute, rpm) at implementation.

Data on the risk of hospitalization were collected through the alert system of the web application. The alerts were determined by the BNP and/or NT pro‐BNP and based on a point system for the calculation of a risk score by the score‐based algorithm. Depending on the calculated risk of hospitalization from the clinical algorithm, patients were categorized into three levels: green (meaning no problem for the patient pursuing usual care and usual telemonitoring), orange (raising awareness among the HCPs and team for the patient to have a consultation (phone call or in‐person, including biological tests)), and red (raising awareness for the team for an emergent contact with the patient and possible hospitalization). Alerts (orange or red) were provided to the care management team by email and/or SMS. A resolved alert from the telemonitoring solution indicated that the HCP acknowledged and validated the alarm whether by managing or not managing the risk; however, not in all cases. Data for the orange and red alerts were collected according to the clinical decision of the HCP in charge, and the delay time from the alert detection to the resolution.

### 2.4 Statistical Analysis

Categorical variables were described in numbers and percentages (%). Continuous variables were described with the mean and standard deviation (SD). Data were filed in Microsoft Excel and were statistically analyzed using Microsoft Excel XLSTAT.

### 2.5 Ethical Considerations

In accordance with French regulations, no ethical declaration was required for the application and approval of patient satisfaction data use and the study conformed to the principles outlined in the Declaration of Helsinki. Patients explicitly gave their consent for the anonymous use of their data.

## 3 Results

In total, 161 patients were included in this study. Most patients were male (82.0%) and the mean age was 62.2 ± 10.7 years (Min: 28, Max: 81) with a mean BMI of 27.9 ± 4.6 kg/m^2^. Most patients (82.0%) had ischemic cardiomyopathy (ICM) (Table 2) with severe LV dysfunction (LVEF% 21.2 ± 6.9) and 38.9% with NYHA ≥ III. Main comorbidities of patients were dyslipidemia (53.4%), a history of coronary artery bypass grafting (CABG) and/or percutaneous coronary intervention (PCI) (44.7%), smoking (42.9%), renal failure (24.8%), diabetes (22.4%) and gastrointestinal bleeding (15.5%) (Table 2).

**TABLE 2:** Characteristics of 161 advanced heart failure patients with LVAD using the Satelia LVAD remote patient monitoring web application in France from March to June 2023 at the inclusion of telemonitoring.

Characteristic	Total, *n*(%)	Mean ± SD [min, max]
Sex (male)	132 (82.0)	
Age range		62.2 ± 10.7 [28, 81]
< 50 years	19 (11.8)	
50–59 years	41 (25.5)	
60–69 years	57 (35.4)	
70–79 years	40 (24.8)	
> 80 years old	4 (2.5)	
Etiology of heart failure		
ICM	132 (82.0)	
DCM	24 (14.9)	
Other	4 (2.5)	
Not available	1 (0.6)	
Therapy type		
BTT	81 (50.3)	
DT	71 (44.1)	
Other	7 (4.3)	
LVEF (%)		21.2 ± 6.9 [10, 41]
TAPSE (mm)		20.2 [7, 21.4]
NYHA Class		
I	6 (3.7)	
II	84 (52.2)	
III	29 (18.0)	
IV	27 (16.8)	
Missing data	15 (9.3)	
Cardiovascular risk factors		
Dyslipidemia	86 (53.4)	
History of CABG and/or PCI	72 (44.7)	
Smoking	69 (42.9)	
Hypertension	66 (41.0)	
Obesity	44 (27.3)	
Comorbidities of interest		
Chronic kidney disease	40 (24.8)	
Diabetes	36 (22.4)	
Gastrointestinal bleeding	25 (15.5)	
Stroke*	16 (9.9)	
Suspected or proven pump thrombosis requiring specific treatment	13 (8.1)	
Obstructive sleep apnea	13 (8.1)	
Depression	10 (6.2)	
PAD	9 (5.6)	
COPD	5 (3.1)	
ICDs	83 (51.6)	
BNP (pg/ml)		581.6 [69, 4,866]
NT pro BNP (pg/ml)		3,625 [180, 33,345]
eGFR (ml/min)		69.5 ± 22.0 [23, 122]
VAD system		
HeartMate III	123 (76.4)	
HeartMate II	18 (11.2)	
HVAD	13 (8.1)	
Jarvik 2000	5 (3.1)	
HeartWare	2 (1.2)	
Pump parameters		
Pump power		22.75 [2, 62]
Pump flow		24.3 [2, 62]
Pump speed, rpm		5, 481 [2300, 9800]
Pulsatility Index		38.33 [2, 94]

Abbreviations: BTT, bridge to transplant therapy; CABG, coronary artery bypass grafting; COPD, chronic obstructive pulmonary disease; DCM, dilated cardiomyopathy; GFR, glomerular filtration rate; ICDs, implantable cardioverter‐defibrillators; ICM, ischemic cardiomyopathy; PAD, peripheral arterial disease; PCI, percutaneous coronary intervention; TAPSE, tricuspid annular plane systolic excursion; rpm, revolutions per minute; VAD, ventricular assist device.

* With supporting CT scans.

Indication for LVAD implantation was mainly ischemic cardiomyopathy (82.0%) and dilated cardiomyopathy (DCM) (14.9%) with 81 patients with 50.3% in BTT (*n* = 81) and 44.1% in DT (*n* = 71). Most patients used the HeartMate III LVAD system (76.4%) and had an implantable cardioverter‐defibrillator (ICD) (51.6%). The mean BNP value was 581.6 pg/mL and the mean NT pro BNP was 3625.0 pg/mL at inclusion (Table 2).

The mean duration of follow‐ups lasted 19.9 months (Min: 1, Max: 45) with 76 patients (47.2%) still undergoing telemonitoring at the study end. Compliance with telemonitoring was high (79.0%). Eighty‐five patients stopped telemonitoring during the study period and the main reasons for cessation were death (mortality rate: 30.6%) (Table 3).

**TABLE 3:** Remote patient monitoring characteristics of 161 LVAD patients using the Satelia LVAD web application in France from March to June 2023.

	Total *n* (%)	Mean ± SD [min, max]
Frequency of digital follow‐ups		
Once a week	156 (96.9)	
Twice a week	4 (2.5)	
Automatic follow‐up	1 (0.6)	
Telemonitoring compliance (questionnaires answered/questionnaires sent)		0.79
DT	71 (44.1)	0.81 ± 0.26 [0.03, 1.0]
BTT	81 (50.3)	0.79 ± 0.25 [0.05, 1.0]
Missing data	9 (5.6)	
Telemonitoring use continued after study	76 (47.2)	
Total Satelia LVAD alerts^†^		
Green alerts	1,553 (13.6)	
Orange alerts	8,265 (72.3)	
Red alerts	1,613 (14.1)	
Reported LVAD infections	47 (29.2)	
INTERMACS adverse events		
Driveline infection during follow‐up	56 (34.8)	
Reason for cessation of telemonitoring (*n* = 85)		
Death	26 (30.6)	
Unsubscribing to the application	22 (25.9)	
Other	19 (22.4)	
Non‐eligible*	9 (10.6)	
Non‐compliance**	6 (7.1)	
Transplant	3 (3.5)	
Mortality rate	26 (30.6)	

* Non‐eligible: patients who no longer needed telemonitoring for their condition.

** Noncompliance: patients who did not answer the questionnaire for 6 weeks.

^†^ Depending on the calculated risk of hospitalization from the clinical algorithm, patients were categorized into three levels: green (meaning no problem for the patient pursuit usual care and usual telemonitoring), orange raising awareness among the HCPs and team for the patient to have a consultation (phone call or in‐person) and red (raising awareness for the team for emergent contact with patient and possible hospitalization). Alerts (orange or red) were provided to the care management team by email and/or SMS.

**FIGURE 2:**
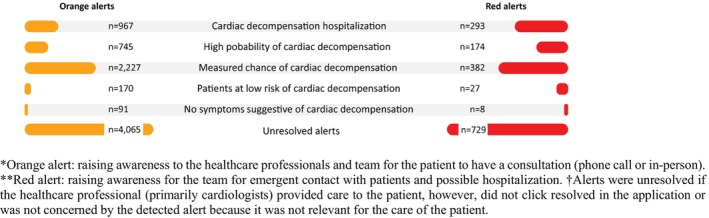
Distribution of orange and red alerts relevance for LVAD patients using the Satelia LVAD remote patient monitoring web application in France, from March to June 2023. [Color figure can be viewed at wileyonlinelibrary.com]

Almost a third of patients had LVAD‐related infections (29.2%) during the follow‐up. Regarding the risk of event/complications detected by the web application, a green status was indicated for 13.6% (*n* = 1553) of alerts, orange alerts were 72.3% (*n* = 8265) and 14.1% (*n* = 1613) were red alerts (Table 3). As shown in Figure 2, among the orange and red alerts, 48.5% of cases were resolved (orange alerts: 50.8% vs. red alerts: 54.8%). The most frequent type of resolved alert was for a measured risk of cardiac decompensation (orange alerts: 2227 vs. red alerts: 382).

## 4 Discussion

To our knowledge, this is the first extensive nationwide study of applying telemonitoring as a structured follow‐up tool specifically for LVAD patients. Over half of LVAD recipients in our cohort were BTT, consistent with previous studies [18, 19]. Our findings show that telemonitoring of adHF patients with LVADs is possible, even in elderly individuals up to 81 years old, and that patient centricity may be achievable by closely monitoring those with persistent, severe symptoms that interfere with and impair daily life activities. Patients were digitally literate and could manage their care alongside HCPs from a distance. Prior studies such as Reiss et al. (2018) [20] have reported that physical consultations for adHF patients in LVAD therapy may last two to three times longer than regular cardiology visitations due to the complexity of the disease, and that telemonitoring can be cost‐effective in terms of reducing human resource use, time and other healthcare costs (e.g., transport costs) [20]. Using qualitative interviews, Walter et al. (2020) described the benefits of telemonitoring for LVAD patients as being safe and enabling earlier detections of complications with quicker interventions in emergency cases [21]. Other cohorts have reported that remote LVAD, including pump flow, power consumption, alarms and symptom reporting can identify hypovolemia, suspected pump thrombosis and driveline infection earlier than routine clinical visitations [20, 22]. Robust randomized trials are limited; however, observational studies such as this present study can support the clinical value of early detection and time effectiveness of outpatient treatment.

The number of telemonitored patients per site in our study varied, and largely depended on the size and capacity of the implanting center's LVAD volume and patient acceptance of having remote follow‐ups. Some centers had an active cohort of over 30 patients. Only digitally literate patients were included, which may have contributed to the site‐to‐site differences and the lower number of patients using the system in specific sites. While non‐enrolled patients had access to the same interventions, activation of care relied on the initiative of patients to contact either by calling the center directly, consulting with their general practitioner (who could then contact the medical team in charge), or through concerns identified during scheduled follow‐ups (whether it was via consultation or day hospitalization). In contrast, telemonitoring improved patient–clinician communication and the connection between patients and involved HCPs, increasing the quality and frequency of follow‐up care and proactive care delivery.

In 2022, the general population with heart failure in France covered approximately 1.1 million individuals with an estimated prevalence of 1.7% and a mortality at 1 year of 34.7% [23]. Though heart failure is well researched, literature on the demographics of LVAD recipients using telemonitoring remains limited with most data based on small monocentric studies. Randomized trials of telemonitoring in the general heart failure population have also produced mixed results. For example, a 2020 trial, comparing telemonitoring to standard monitoring for all heart failure patients not specific to LVAD, showed no significant reductions for all‐cause deaths or unplanned hospitalizations [24], although patients with more severe heart failure (LVEF< 40%) benefited more [24]. A systematic meta‐analysis covering 92 studies from 1996 to 2022, similarly suggested that adHF patients profit more from invasive forms of hemodynamic monitoring than telehealth solutions and that telemonitoring systems were more catered to patients with NYHA class I to III [25]. Nevertheless, smaller LVAD‐specific studies indicate a meaningful clinical impact. Mariani et al. (2021) [18] reported that their telemonitoring program using phone calls and a 24/7 emergency LVAD line, for 156 patients in Germany detected 14.6% of cases that needed further evaluation based on telemonitored alerts [18]. Literature reviews also highlight that remote LVAD monitoring can detect adverse events earlier than routine care, allowing timely outpatient interventions [14, 20, 23]. Recent small‐scale studies have also explored prototype algorithm‐driven analysis of LVAD pump telemetry, symptom reports and other sensors to predict complications before symptoms arise [14, 20, 26].

Our study used the alert system of Satelia LVAD which was able to specifically distribute the risk of hospitalizations into categories and may have improved event detection compared to other forms of monitoring. However, alarm fatigue and clinically irrelevant alerts due to weighting remain recognized challenges in LVAD telemonitoring [14, 20]. Many alerts in our system were resolved clinically but were not logged as adverse events. This may highlight the need for better filtering and prioritization. Recommendations may include dedicated triage teams, individualizing alarm thresholds, and protocols to integrate alerts efficiently into clinical workflows [14, 20].

The relationship between patients and HCPs in LVAD care is complex and continues to evolve with advances in technology and care delivery models. LVAD management requires intensive, ongoing shared decision‐making and therapeutic education, potentially beyond the traditional follow‐up processes in heart failure care. Clinician workloads to maintain and manage high volumes of telemetry and alerts, especially without defined triage roles and protocols for adHF patients, increase cognitive and administrative burden [20]. Patients and caregivers must learn device maintenance, alarm interpretation, driveline care and symptom recognition which expand the scope of responsibilities for HCPs beyond routine cardiology care [27, 28]. Caregiver involvement is also a central component in LVAD care management and affects both patient outcomes and the relationship between HCPs and patients. Multiple studies have shown that caregiver capacity, strain and characteristics influence patient QoL, as well as therapy adherence and the continuity of care. Consequently, caregivers become active intermediaries in communication and decision‐making with clinical teams [29, 30]. To achieve optimal outcomes, telemonitoring systems must be fully integrated into clinical workflows and individualized alarm thresholds, as well as health records integration, secure data governance and algorithmic filtering [14]. The design and implementation of such systems would require co‐development with HCPs and patients to ensure trust and usability and support sustained long‐term LVAD care.

Although this study did not directly measure clinical outcomes, existing literature demonstrates that patients using telemonitoring report higher satisfaction with engagement and communication compared with standard care [16, 31]. More importantly, telemonitoring promotes earlier recognition of complications which allows timely requests and interventions such as diuretics, antibiotics, and complementary diagnostic evaluations [20, 21, 23]. For example, photo‐based monitoring has shown value in early detection of LVAD‐related infections. Daily weight and symptom reporting can also guide prompt responses to heart failure decompensation. The capacity to intervene earlier, not only reduces the risk of heart failure severity but may provide easier access to treatment outside of hospital settings, particularly for treatment initiation in outpatient settings before symptoms worsen even when hospitalization is required. Therefore, time‐sensitive care is a key advantage of telemonitoring in heart failure care management which is particularly relevant when facilitating early detection and intervention for changes in clinical status in frail or elderly patients with infections or signs of HF deterioration [14, 20, 22]. Another benefit is the reduced travel and clinical burden of patients. Telemonitoring can reduce the need for frequent in‐person checks and can enable more targeted visitations. A national‐scale, real‐world, propensity‐weighted cohort study using the SNDS French database in which remote monitoring with Satelia Cardio was used, was associated with lower all‐cause mortality, emergency visits, and length of stay at the hospital [32]. These results were consistent across age, even in patients older than 80 years. Similarly, Walter et al. (2019) reported improved convenience and patient satisfaction toward the use of telemonitoring [21].

Regarding limitations, the retrospective and observational design as well as missing data may affect the generalizability of the study. We did not have sufficient data to compare device subgroups such as the Heart Mate III versus HVAD for further analysis. French data privacy legislations prevented the integration of direct LVAD hardware telemetry into the telemonitoring system which may have limited our findings on physiological precisions. The web application used declarative patient‐reported monitoring as opposed to direct device interrogation which may have been less accurate than implantable sensors/machine monitoring such as the Cardio‐MEMS‐type system, even though it is noninvasive and lower in cost. Follow‐up of LVAD patients was limited to death, heart transplantation or the cessation of follow‐up; future studies should provide hospitalization rates, indications and complication profiles that could facilitate multidisciplinary team follow‐ups as well as describe predictive factors of adverse events based on telemonitoring. Research on adapting the follow‐up of patients may be necessary to optimize clinician workflows, integrate telemonitoring data into health records and define standardized alert triage and escalation pathways, particularly at initial hospital discharge.

## 5 Conclusions

Telemonitoring may potentially be a feasible follow‐up tool to provide feedback for LVAD patients. Our study showed that telemonitoring may particularly be used when detecting early risks of complications. Future randomized studies on specific prospective evaluations such as survival and QoL should be conducted in adHF.

## Author Contributions

Clément Delmas: supervisor, conception, interpretation of the data, data collection, writing, and validation of the manuscript. Anne‐Sophie Simoni, Céline Goeminne, Aude Boignard, Anne‐Céline Martin, Fabrice Vanhuyse, Erwan Flecher, and Catherine Nafeh‐Bizet, Romain Itier, and Pascal Battistella: data collection and reviewing of the manuscript. Nicolas Pages: conception, coordination, writing and reviewing of the manuscript. Sophie Nisse‐Durgeat: conception, coordination writing, and reviewing of the manuscript. Karine Nubret‐le‐Coniat: supervisor, data collection and reviewing of the manuscript. All authors reviewed and approved the final manuscript.

## Conflicts of Interest

The authors declare no conflicts of interest.

## Supporting information


**Table S1:** Overview of the Satelia LVAD questionnaire provided to patients for telemonitoring

## Data Availability

The data that support the findings of this study are available from the corresponding author upon reasonable request.
