# Supplementary material for: Follow‐Up of Left‐Ventricular Assist Device Patients With Telemonitoring: A National Retrospective Multicentric Study on the Satelia LVAD Web Application
Source: Artif Organs. 2025 Nov 8;50(2):250–8. doi: 10.1111/aor.70038 (PMC12993257; doi:10.1111/aor.70038)
Supplement: Supplementary file 1 — Table S1: Overview of the Satelia LVAD questionnaire provided to patients for telemonitoring [file AOR-50-250-s001.docx]

**Supplementary Material**

**Supplementary Table 1.** Overview of the Satelia® LVAD questionnaire provided to patients for telemonitoring

| Question | Item type | Answers |
| --- | --- | --- |
| Baseline parameters |  |  |
| *Sex* | Binary | Male/Female |
| *Age* | Multiple choice question | <50 years, 50 to 59 years, 60 to 69 years, 70 to 79 years, >80 years old |
| *Etiology of heart failure* | Multiple choice question | Ischemic cardiomyopathy (ICM), dilated cardiomyopathy (DCM), Other, Not available |
| Circulatory support device |  |  |
| *Do you have an ICD?* | Binary | Yes/No |
| *NYHA Class* | Free response format | - |
| *LVEF (%)* | Free response format | - |
| *VAD system* | Multiple choice question | *HeartMate III****,*** *HeartMate II****,*** *HVAD****,*** *Jarvik 2000****,*** *HeartWare* |
| Baseline mechanical parameters |  |  |
| *RPM* | Free response format | - |
| *Pulsatility Index* | Free response format | - |
| Baseline laboratory parameters | Free response format | - |
| *BNP (pg/ml)* |  |  |
| *NT-proBNP (pg/ml)* | Free response format | - |
| *LDH* | Free response format | - |
| *Creatinine* | Free response format | - |
| *Serum natremia* | Free response format | - |
| *eGFR (ml/min)* | Free response format | - |
| Baseline RV function parameters |  |  |
| *Tricuspid S wave* | Free response format | - |
| *TAPSE (mm)* | Free response format | - |
| *Isotopic RVEF* | Free response format | - |
| Parameters during telemonitoring |  |  |
| *What is your International Normalized Ratio (INR)?* | Free response format | - |
| *What is your temperature?* | Free response format | - |
| *What is your systolic/diastolic/ mean blood pressure (BP)? (in mmHg)* | Free response format | - |
| *Since your last doctor's visit, have you experienced any of the following symptoms?* | Multiple answer question | Blurred vision/blood in urine/blood in stool/black stool/shortness of breath at rest/dizziness/headache /none of these symptoms |
| *Does the driveline orifice exhibit any of the following symptoms?* | Multiple choice question | Redness/discharge/pain/none of these symptoms |
| Pump parameters |  |  |
| *What is the pump power? (in W)* | Free response format | - |
| *What is the flow rate? (in L/min)* | Free response format | - |
| *Have there been any recent alarms on your Satelia® LVAD device?* | Binary | Yes/No |

VAD; ventricular assist device, rpm; revolutions per minute, LDH; lactate dehydrogenase, ICD; implantable cardioverter-defibrillator, GFR; glomerular filtration rate, RV; right ventricular, TAPSE; tricuspid annular plane systolic excursion.
